# Naïve T Cell Quiescence in Immune Aging

**DOI:** 10.20900/agmr20210015

**Published:** 2021-06-26

**Authors:** Claire E. Gustafson

**Affiliations:** Division of Immunology and Rheumatology, Department of Medicine, Stanford University, Stanford, CA 94304, USA;

**Keywords:** immune aging, cellular homeostasis, tissue niches, stem cells, differentiation

## Abstract

Naïve T cells are critical for protection against emerging viral and bacterial infections. However, the ability of these cells to elicit effective long-term immune responses declines with age and contributes to increased disease susceptibility in older individuals. This decline has been linked with the breakdown of cellular quiescence that causes partial differentiation of naïve T cells with age, but the underlying mediators of this breakdown are unclear. Comparisons to stem cell quiescence in mice and man offer insight into naïve T cells and aging. However, the utilization of single cell technologies in combination with advances in the biology of human tissue aging is needed to provide further understanding of naïve T cell complexity and quiescence breakdown with age.

## INTRODUCTION

A key feature of age-related immune erosion (termed “immune aging”) is the loss of naïve T cells [1,2]. This loss is often attributed to the involution of thymus during adulthood however naïve T cells can be maintained for decades by homeostatic proliferation within lymph nodes and secondary lymphoid tissues [1] Naïve cell loss is instead caused by a breakdown in peripheral homeostasis during the aging process. Naïve T cell homeostasis is multi-faceted, requiring both cell survival and the retention of a quiescent state. Recent studies in humans highlight that naïve cells not only decline numerically in lymph nodes [3], but they also break quiescence, acquiring a distinct, partially differentiated state during aging [4–6]. Here, we will discuss these novel findings and potential age-specific mediators of these changes. We highlight the striking similarities between stem cell and naïve T cell quiescence and propose a model in which aging lymph node niches fail to maintain a ‘deep’ quiescent state of naïve T cells but instead drive them towards an ‘shallow’ quiescence state we observe experimentally as partial differentiation.

## QUIESCENCE AND NAÏVE T CELL PARTIAL DIFFERENTIATION

Stem cell quiescence is a reversible state of growth arrest that plays an important role in tissue homeostasis and regeneration. Recent work in the area of stem cell biology has established that quiescence is not a passive process but is actively maintained by transcriptional and post-transcriptional regulation, including chromatin modification and microRNA-mediated gene repression [7,8]. Notably, there are distinct levels of stem cell quiescence, ranging from ‘deep’ to ‘shallow’ that correlated with more rapid responses and altered functional capacity in both mice and man [9,10]. A transition from deep to shallow state of quiescence is driven by signals derived from nearby or distant tissue injury, whereas the exit from quiescence occurs when there is local tissue injury. Stem cells can cycle between different states of quiescence depending on their local interactions with other cells, extracellular matrix and cytokines. During aging, stem cell quiescence is dysregulated, leading to cell death, cellular senescence and/or altered differentiation [11].

Biologically, naïve T cells are relatively similar to quiescent stem cells, particularly in their high pluripotency and proliferative potential. However, unlike stem cells, the extracellular cues for exit from quiescence are unique to naïve T cells. These cells classically retain a quiescence state until they encounter a specific antigen within their local lymph node niche. Upon direct antigen activation, naïve T cells exit quiescence, rapidly proliferate and can differentiate into numerous functional states depending on numerous factors including the local cytokine and cellular milieu. In turn, the regulation of activation and the maintenance of cellular quiescence in T cells is extremely important for immune homeostasis, as its failure can lead to significantly perturbed immunity, such as autoimmune disease, cancer or increased infection [12]. In aging, proliferation capacity of naïve T cells appears intact however pluripotency is diminished; naïve T cells from older individuals display reduced ability to form memory and skewing of subset polarization [13,14]. These data collectively suggest a partial breakdown in cellular quiescence.

**Figure 1.**
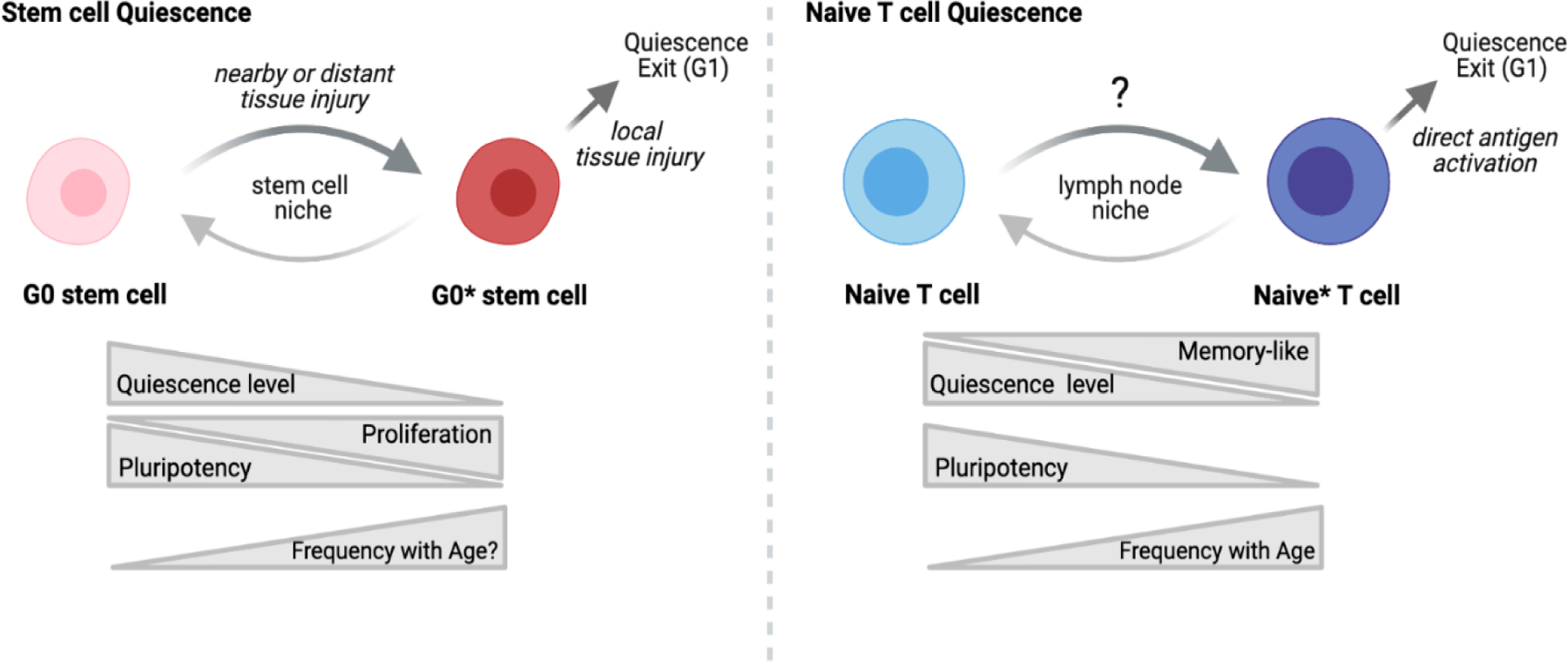
Model of Naïve T cell Quiescence with Aging. During aging, naïve T cells (Naïve) become partially differentiated (Naïve*), acquiring some features of memory T cells while retaining a phenotypically naïve state. In this memory-like naïve state, aging T cells demonstrate reduced pluripotency with altered subset differentiation post-activation. These features are similar to that observed in stem cells, where the level of quiescence (deep G_0_ (G0) → shallow G_0_ (G0*)) dictates their proliferative and differentiation potentials. Thus, a model arises in which naïve T cells in young adults are maintained in a deep quiescent state whereas naïve T cells in older individuals receive altered signaling from the aging lymph node microenvironment that drives the cells towards a shallower state of quiescence. Image created with BioRender.com.

Growing evidence demonstrates that the naive T cell population epigenetically and transcriptionally shift towards a more memory-like state with age [4–6]. These memory-like features include a chromatin landscape bias towards memory cell features (e.g., increased accessibility of BATF) as well as global upregulation of differentiation-related microRNAs (e.g., mir-146a). Possible causes of this phenotypic shift are 2-fold: (1) selection of cells with a fitness advantage or (2) adaptation of cells to an aging tissue niche. In mice, an adaptation scenario is mathematically favored, where cells adapt to their environment, acquiring survival and/or proliferative advantage with age [15]. Notably, fate mapping studies have found that the naïve T cells are epigenetically primed for different functionality based on the animals’ age when the cell was generated [16,17]. As humans lose the ability to make new naïve T cells via thymic output later in life whereas mice do not [18,19], the translatability of this age-dependent naïve T cell heterogeneity is unclear. However, recent studies using single cell analysis of human naïve T cell populations suggests that an adaptation/conversion scenario is more likely, as naïve T cells from older individuals can reacquire young-like features under certain in vitro growth conditions (unpublished data [20]). In light of the current knowledge on stem cell biology, we propose that the shift towards a memory-like state in naïve cells with age is an adaption to an aging lymph node niche, in which naive T cells shift from a state of long-term, deep quiescence into a shallower one via age-related extracellular signals (Figure 1).

## REGULATORS OF NAÏVE T CELL QUIESCENCE AND ITS BREAKDOWN WITH AGE

During aging, numerous changes occur in stem cell niches that contribute to stem cell-intrinsic dysfunction and loss of quiescence (e.g., increased inflammatory cytokines, altered extracellular matrix composition) [21]. Thus, the partially differentiated state of naïve T cells could similarly be driven by age-related changes in local lymph node niches. In youth, naïve T cell homeostasis is maintained with secondary lymphoid tissues (SLT) (i.e., lymph nodes) by specialized stromal cells, fibroblastic reticular cells (FRCs). In animal models, aging SLTs exhibit a collapse of stromal networks, an increase in fibrosis and reductions in homing chemokines levels [22–25], suggesting FRC dysfunction during aging may be associated with naïve T cell quiescence breakdown.

FRCs classically maintain homeostasis by secretion of the essential survival cytokine IL-7. However, multiple studies on IL-7 and age have ruled out the differential production of IL-7 by FRCs as a cause of homeostatic failure in aged naïve T cells in both mice and man [25–27]. FRCs also secretes a range of other soluble factors (e.g., prostaglandin E2) that have been shown to actively suppress TCR-induced cellular differentiation [28–30]. Thus, active inhibition of differentiation signals in naïve T cells may be required to maintain a deep quiescence state and long-term survival. This idea would be similar to stem cell homeostasis where extracellular cues, such as Notch and Wnt signaling, help reenforce a quiescence state [8]. Indeed, human FRCs can directly suppresses naïve T cell proliferation and memory differentiation via the combination of factors such as TGF-beta and adenosine [31]. Adenosine and TGF-beta signaling also helps maintain naïve T cell quiescence in mice [32,33]. Whether such inhibitory factors also play a functional role in maintaining human naïve T cell quiescence and/or mediate the transition from deep into shallow quiescent states with age remains to be determined.

## CONCLUSIONS

It is clear that naïve T cells undergo a breakdown in quiescence with age. The transition into a ‘shallow’ quiescent state observed in stem cells shares similarities with the partially differentiated features we find in naïve T cell aging. However, the casual mechanisms for this change in humans remain elusive. The use of single cell technologies in mice has led to many important immunological insights. They have demonstrated that tissue niches can have powerful effects on dictating T cell fate [34]. Moreover, although relatively ignored, structural cells (i.e., stroma, epithelium, endothelium) can play significant roles in immune homeostasis and disease, having high amounts of cellular crosstalk with immune cells across organs [35]. Interrogation of human SLTs across age using newer single cell techniques such as TEA-seq (allows interrogation of chromatin landscape, RNA and protein expression in a single cell) [36] and spatial genomics (allows cellular identification and functionality in the context of tissue localization) [37], in combination with development of baseline cellular atlases similar to the Tabula Muris Senis Atlas [38], would provide significant insight into the heterogeneity of human naïve T cell quiescence and interactions within lymph node niches that are essential for control of quiescence and its breakdown with age. Moreover, the development and utilization of novel models systems, such as human lymph node organoids [39], is needed to better enhance translatable, mechanistic studies in human T cells and to ultimately help to improve overall T cell responses to infections and vaccination in the growing population of older individuals [40].
